# Chemical Composition, Antioxidant Potential, and Nutritional Evaluation of Cultivated Sorghum Grains: A Combined Experimental, Theoretical, and Multivariate Analysis

**DOI:** 10.3390/antiox12081485

**Published:** 2023-07-25

**Authors:** Simona Jaćimović, Biljana Kiprovski, Petar Ristivojević, Dušan Dimić, Đura Nakarada, Biljana Dojčinović, Vladimir Sikora, Nemanja Teslić, Nebojša Đ. Pantelić

**Affiliations:** 1 Institute of Field and Vegetable Crops, National Institute of the Republic of Serbia, Maksima Gorkog 30, 21000 Novi Sad, Serbia; simona.jacimovic@ifvcns.ns.ac.rs (S.J.); biljana.kiprovski@ifvcns.ns.ac.rs (B.K.); vladimir.sikora@ifvcns.ns.ac.rs (V.S.); 2 Department of Analytical Chemistry, Faculty of Chemistry, University of Belgrade, Studentski trg 12-16, 11158 Belgrade, Serbia; ristivojevic@chem.bg.ac.rs; 3 Faculty of Physical Chemistry, University of Belgrade, Studentski trg 12-16, 11000 Belgrade, Serbia; ddimic@ffh.bg.ac.rs (D.D.); djura@ffh.bg.ac.rs (Đ.N.); 4 Department of Chemistry, Institute of Chemistry, Technology and Metallurgy, University of Belgrade, Studentski trg 14, 11000 Belgrade, Serbia; bmatic@chem.bg.ac.rs; 5 Institute of Food Technology, University of Novi Sad, Bulevar cara Lazara 1, 21000 Novi Sad, Serbia; nemanja.teslic@fins.uns.ac.rs; 6 Department of Chemistry and Biochemistry, Faculty of Agriculture, University of Belgrade, Nemanjina 6, 11080 Belgrade, Serbia

**Keywords:** sorghum, chemical composition, antioxidant activity, nutritional quality, multivariate analysis, DFT

## Abstract

Sorghum grain (*Sorghum bicolor* L. Moench) is a gluten-free cereal with excellent nutritional value and is a good source of antioxidants, including polyphenols, as well as minerals with proven health benefits. Herein, the phenolic composition, elemental profile, and antioxidant activity of sixteen food-grade sorghum grains (**S1**–**S16**) grown under agroecological conditions in Serbia were determined. Nine phenolic compounds characteristic of sorghum grains, such as luteolinidin, 5-methoxyluteolinidin, luteolidin derivative, luteolidin glucoside, apigeninidin, 7-methoxyapigeninidin, apigeninidin glucoside, and cyanidin derivative, were quantified. The antioxidant potential of the analyzed sorghum grains was evaluated by UV/Vis (DPPH, ABTS, and FRAP) and Electron Paramagnetic Resonance spectroscopy (hydroxyl and ascorbyl radical scavenging assays). The content of macro- and microelements was determined by Inductively Coupled Plasma Optical Emission spectroscopy. Theoretical daily intakes of selected major and trace elements were assessed and compared with the Recommended Daily Allowance or Adequate Intake. Sample **S8** had the highest amount of phenolic compounds, while **S4**, **S6**, and **S8** exhibited the strongest antioxidative potential. The sorghum studied could completely satisfy the daily needs of macro- (K, Mg, and P) and microelements (Se, Zn, Fe). Pattern recognition techniques confirmed the discrimination of samples based on phenolic profile and elemental analysis and recognized the main markers responsible for differences between the investigated samples. The reaction between hydroxyl radicals and luteolinidin/apigeninidin was investigated by Density Functional Theory and thermodynamically preferred mechanism was determined.

## 1. Introduction

Sorghum (*Sorghum bicolor* L. Moench) is the fifth most widely grown cereal in the world, cultivated globally due to its adaptability to high temperatures and lower rainfall [1,2]. As global demand for greater productivity in low-quality soils increases, sorghum is ideally suited to meet this need. It is a sustainable crop that requires significantly less water than other crops and is adaptable to drought conditions and high temperatures [3,4]. 

The nutritional composition of sorghum grains is comparable to maize and rice in terms of protein, starch, and mineral content [5]. In addition to its outstanding agronomic advantages, sorghum is gaining interest due to its beneficial nutritional and functional properties. It is a gluten-free grain that provides a solution for people with gluten intolerances [6,7,8], but it is also a very rich source of unique phenolic compounds and dietary fibers [9,10,11,12]. Abundant nutraceuticals, along with the fact that sorghum is a gluten-free grain, make it an attractive alternative to other cereals, such as wheat, rye, and barley. With a rising shift in consumers’ demand toward developing functional foods, sorghum has the potential to be used as a new food ingredient. The grain is a good starch source, with approximately 65 to 80% of its dry weight [13]. Apart from that, the advantage of sorghum is its protein content of an average of 7 to 15% [13,14]. From a nutritional point of view, the value of sorghum grains could be considered slightly inferior compared to other cereal grains based on lower protein and starch digestibility. The lower starch digestibility reported for sorghum is not an essential property of the starch granules themselves but appears mainly to be a consequence of the interactions of starch with the protein matrix, as well as with condensed tannins and flavonoids [15]. 

Although sorghum is an important source of nutrients, it contains the hard to digest protein kafirin and a low content of the essential amino acid lysine [16]. Proteins and mineral nutrients are known to have a close relationship in biological systems [17]. Since cereals can be an important source of essential nutrients due to their large daily intake, knowing their composition is very important. Mineral nutrients play a fundamental role in the proper functioning of any living organism. Sorghum contains a substantial amount of various minerals, with bioavailability ranging from low (less than 1%) to more than 90% for Na and K [18,19], so it has the potential to provide a significant concentration of minerals in the human diet. However, the elemental composition may vary depending on the conditions of cultivation and the geographical origin of samples.

Health-promoting polyphenols are highly present in sorghum grains [9,10], making them interesting cereals as a source of bioactive compounds. Their composition and concentration vary depending on the genotypes and production environment [20,21,22]. Anthocyanins found in some sorghum varieties are of notable interest since they are unique to sorghum grain and potentially have powerful antioxidant potential [23,24,25]. In addition to their antioxidant and antimicrobial properties, dominantly present 3-deoxyanthocyanins (3-DXA), apigeninidin, and luteolinidin provide an attractive color in certain sorghum varieties [23]. The antioxidant activity of sorghum chemical compounds has been associated with many beneficial properties for human health, such as oxidative stress reduction, anticancer, antidiabetic, and anti-inflammatory activity [26,27]. Hong et al. [28] recently reported a link between antioxidant activity, tannin content, and anti-inflammatory potential. 

The present study aimed to determine and compare the phenolic composition, macro- and microelement content, and antioxidant activity of preselected sorghum grain varieties (16 genotypes) suitable for human consumption as functional ingredients of anti-allergy food. Principal component analysis (PCA) and hierarchical cluster analysis (HCA) have been applied to phenolic and elemental content to identify the most promising group of samples with a higher content of antioxidants and minerals. In addition, a theoretical assessment of the nutritional value of the studied grains, expressed via the elemental composition, was performed in order to determine their potential as a source of macro- and microelements for human nutrition. Density functional theory and Natural Bond Orbital analysis were applied to investigate the structure, stability, and thermodynamically preferred HO^•^-scavenging mechanism of luteolinidin and apigeninidin.

## 2. Materials and Methods

### 2.1. Samples

Sorghum grain samples were obtained from the Institute of Field and Vegetable Crops, Novi Sad, National Institute of the Republic of Serbia. Sixteen varieties of sorghum with high genetic variability, including varieties of the yellow, red, and brown pericarp, were analyzed and marked as **S1**–**S16**. The samples were cultivated under the same conditions at the Department of vegetable and alternative crops (Bački Petrovac N 50°21′; E 39°56′) in a conventional farming system for the production of commercial genotypes of the Institute in 2020. The samples’ names, genotypes, and original collections are listed in the Supplementary Material, Table S1.

### 2.2. Determination of 3-Deoxyanthocyanidin Composition

In order to determine the composition of phenolic compounds, sorghum extracts were obtained after grinding whole-grain sorghum in a mill (IKA A11 basic analytical mill). The milled samples were passed through a 0.5 mm sieve, vacuum-sealed, and stored in a refrigerator until analysis. The extraction technique was adopted as the most often used technique from previous studies by research groups [29,30,31]. Briefly, the samples were weighed (0.5000 ± 0.0001 g) on an analytical balance (Denver Instrument, Inc., Bohemia, NY, USA) and extracted with 10 mL of MeOH as an extraction agent. Extraction was performed in an ultrasonic bath for 45 min with the addition of ice to prevent heating of the samples. Then, the extracts were centrifuged at 1960× *g* for 10 min, and the obtained supernatants were transferred to separate tubes using an automatic pipette and used for further work. The extraction was performed in three replicates per sample. 

The content of 3-deoxyanthocyanidins was determined according to the modified method reported by Xiong et al. [32]. Briefly, previously prepared extracts were evaporated under the N_2_ atmosphere, reconstituted with methanol, and filtered through a regenerated cellulose syringe filter (0.45 µm). The prepared samples were injected into the HPLC/DAD system (1200 Series, Agilent, Santa Clara, CA, USA) with a Zorbax Eclipse XDB-C18 (4.6 mm× 50 mm × 1.8 µm) analytical column. Separation of 3-deoxyanthocyanidins was performed using a 1% aqueous formic acid solution as mobile phase A and acetonitrile as mobile phase B. The gradient for mobile phase A was set as follows: 95% (0 min), 95–92% (5 min), 92–79% (35 min), 79–65% (40 min), 65–95% (45 min), and 95% (49 min). Other parameters, such as flow rate, DAD wavelength, and injection volume, were set as described by Xiong et al. [32]. The calibration curves of luteolinidin (R^2^ = 1) and apigeninidin (R^2^ = 0.9997) were constructed with 5 calibration points of analytical standards in the concentration range 0.75–15 µg mL^−1^. The results were expressed as micrograms of polyphenols per gram of sample (µg g^−1^).

### 2.3. Determination of Antioxidant Activity

#### 2.3.1. DPPH Radical Scavenging Assay

The antioxidant activity of the tested MeOH extracts was determined using the free radical 2,2-diphenyl-1-picrylhydrazyl (DPPH) according to the method described by Brand-Williams et al. [33]. The concentration of the working solutions was 0.05 g mL^−1^. Briefly, 100 µL of the samples was mixed with 4 mL of DPPH radical solution (150 μM), and the tubes were incubated in the dark for 30 min. Afterward, the absorbance was measured at 515 nm on a UV-Vis spectrophotometer (Du-8200 Single Beam UV/Vis spectrophotometer, Shanghai, China). DPPH radical scavenging activity was expressed as µmol Trolox-equivalent antioxidant capacity per gram of dry weight of the sample (µmol TE g^−1^ DW). The calibration curve of Trolox was constructed with six calibration points in the concentration range 0–600 µg mL^−1^.

#### 2.3.2. ABTS Assay

The ABTS assay is a widely used spectrophotometric method for the evaluation of antioxidant capacity, based on the quenching of a stable-colored radical (ABTS^•+^), indicating the radical scavenging ability of antioxidants. The ABTS assay was performed using the method described by Munteanu and Apetrei, with slight modifications [34]. Briefly, the ABTS^•+^ reagent was prepared by mixing equal volumes of 5 mM ABTS and 2.45 mM K_2_S_2_O_8_ solution. The resulting mixture was kept at room temperature in the dark for 24 h, and then the obtained solution was diluted with ethanol until the measured absorbance at 734 was close to 0.8. The volume of 50 µL of the extract solution was mixed with 4 mL of ABTS^•+^ reagent, and the absorbance of each sample was measured after 30 min at 734 nm (Du-8200 Single Beam UV/Vis spectrophotometer, Shanghai, China). The results were expressed as µmol Trolox-equivalent antioxidant capacity (TE) per gram of dry weight of the sample (µmol TE g^−1^ DW). The calibration curve of Trolox was constructed with six calibration points in the concentration range 0–600 µg mL^−1^. 

#### 2.3.3. FRAP Assay

The ferric-reducing antioxidant power (FRAP) assay was performed using the method described previously [35]. Briefly, the extract solution (100 µL, 0.05 g mL^−1^) was mixed with 300 µL of distilled water and 3 mL of FRAP reagent. The FRAP reagent was prepared by mixing 2.5 mL of 10 mM 2,4,6-tripyridyl-*s*-triazine (TPTZ), 2.5 mL of 20 mM FeCl_3_ × 6H_2_O, and 25 mL of 300 mM acetate buffer (pH 3.6). The absorbance was measured after 40 min at 593 nm (Du-8200 Single Beam UV/Vis spectrophotometer, Shanghai, China). The results were expressed as µmol Trolox-equivalent antioxidant capacity per gram of dry weight of the sample (µmol TE g^−1^ DW). The calibration curve of Trolox was constructed with six calibration points in the concentration range 0–600 µg mL^−1^.

#### 2.3.4. Hydroxyl Radical (HO^•^) Scavenging Assay by Electron Paramagnetic Resonance (EPR) Spectroscopy

To study the scavenging effect of sorghum extracts towards HO^•^ radicals, a solution consisting of MeOH sorghum extracts in the presence of a Fenton reaction containing DEPMPO (5-diethoxyphosphoryl-5-methyl-1-pyrroline-*N*-oxide) spin-trap was used [36,37]. This spin-trap was selected for its good selectivity and long DEPMPO/OH^•^ spin-adduct half-life [38]. The sample of the total volume of 29 µL which contained 26 µL of deionized water, 2 µL of H_2_O_2_ (final concentration 0.35 mM), and 1 µL of DEPMPO (final concentration 3.5 mM) were mixed together with 1 µL of FeSO_4_ (final concentration 0.15 mM), transferred into the gas-permeable Teflon tube which was inserted into the EPR resonator (Bruker ELEXSYS-II E540) and EPR signal of DEPMPO/OH spin-adduct was recorded after 2 min using the following experimental parameters: center field 3500 G, microwave power 10 mW, microwave frequency 9.85 GHz, modulation frequency 100 kHz, modulation amplitude 1 G. The control recordings were made by adding 1 µL of MeOH instead of the sorghum extracts. The antioxidant activity of the sorghum extracts (AA) was calculated using the formula:

AA = 100 × (Ic − Ia)/Ic (%)(1)

where Ic and Ia refer to the average intensity of the two most intense signals in the low field (denoted by blue circle in the Results section) of EPR spectra of control and sorghum samples, respectively. 

#### 2.3.5. Ascorbyl Radical (Asc^•^) Scavenging Assay by Electron Paramagnetic Resonance (EPR) Spectroscopy

To determine the scavenging potential of sorghum extracts toward ascorbyl radicals, the EPR signal of ascorbyl radical (Asc^•^) in DMSO (dimethyl sulfoxide) solution was recorded in the system containing sorghum extracts in MeOH using the previously developed procedure [37,39]. The solution containing 10 µL of sorghum extract in MeOH, 5 µL of water solution of ethylenediaminetetraacetic acid, EDTA (final concentration 0.2 mM), and 0.5 µL of water solution of FeCl_3_ (final concentration 7 µM) were mixed to form the Fe(III)-EDTA complex, followed by the addition of 42 µL of DMSO. A volume of 2.5 µL of DMSO solution with ascorbic acid (final concentration 0.2 mM) was added into the mixture, and 30 µL of it was transferred into the gas-permeable Teflon tube. This tube was placed into a quartz EPR cuvette, which was inserted into an EPR resonator, and the EPR signal was recorded after 2 min using the following experimental parameters: center field 3500 G, microwave power 10 mW, microwave frequency 9.85 GHz, modulation frequency 100 kHz, and modulation amplitude 1 G. Control recordings were made by adding 10 µL of MeOH instead of the sorghum extracts. The antioxidant activity of the sorghum extracts was calculated using the previously described formula.

### 2.4. Content of the Major and Trace Elements

The microwave digestion (total mineralization) of the samples of sorghum was performed on an Advanced Microwave Digestion System (Ethos 1, Milestone, Italy) using HPR-1000/10S high-pressure segmented rotor. Pressure-resistant PTFE vessels (volume 100 mL), which were equipped with QS-50 Quartz inserts, were used. Samples were weighed precisely (0.5000 ± 0.0001 g), placed in the quartz insert, and mixed with 4.5 mL HNO_3_ (65%, Suprapur^®^) and 0.5 mL H_2_O_2_ (30%, Suprapur^®^) (both of Merck KGaA, Darmstadt, Germany). The temperature was gradually raised with microwave power (0–1000 W): linearity from 25 to 180 ºC in the first 15 min, remained at 180 °C in the next 20 min, and then decreased rapidly to room temperature. After cooling, without filtration, the solution was diluted to a fixed volume of 25 mL in a volumetric flask with ultrapure water. Ultrapure water with a conductivity of 0.05 µS cm^−1^ was prepared using a Barnstead™ GenPure™ Pro (Thermo Scientific, Germany). Twenty-two elements were analyzed using the optical emission spectrometry with inductively coupled plasma (ICP-OES) technique: aluminum (Al), arsenic (As), boron (B), barium (Ba), calcium (Ca), cadmium (Cd), cobalt (Co), chromium (Cr), copper (Cu), iron (Fe), potassium (K), lithium (Li), magnesium (Mg), manganese (Mn), sodium (Na), nickel (Ni), phosphorus (P), lead (Pb), sulfur (S), selenium (Se), strontium (Sr), and zinc (Zn).

#### ICP-OES Measurement

The contents of major and trace elements in the solution samples were determined by inductively coupled plasma optical emission spectrometry, ICP-OES (iCAP 6500 Duo ICP, Thermo Fisher Scientific, Cambridge, United Kingdom). The external calibration solutions were made from three certified plasma standard solutions: Multi-Element Plasma Standard Solution 4, Specpure^®^, 1000 µg mL^−1^ (Alfa Aesar GmbH & Co. KG, Karlsruhe, Germany); SS-Low Level Elements ICV Stock; and ILM 05.2 ICS Stock 1 (both of VHG Labs, Inc., Part of LGC Standards, Manchester, NH, USA). Quality control was carried out using blank samples, matrix-matched calibration solutions, and triplicate analyses of each sample. The reliability of the measurements was approved by a relative standard deviation (RSD) < 1%. The limit of detection (LOD) was in the range of 0.05–1.5 µg L^−1^ (0.0025–0.075 mg kg^−1^), and the limit of quantification (LOQ) was in the range of 0.1–5 µg L^−1^ (0.005–0.25 mg kg^−1^) in solutions of totally mineralized samples. The analytical process quality control (QC) was performed using two certified reference materials (CRMs) of fish protein for trace metals DORM 4 (NRCC, National Research Council Canada, Ottawa, Ontario Canada) and EPA Method 200.7 LPC Solution (ULTRA Scientific, North Kingstown, RI, USA). The recovery of measured concentrations with certified values was 98–103%. The concentrations of elements in the samples were expressed as mg kg^−1^ (ppm).

### 2.5. Determination of the Thermodynamically Preferred Mechanism of Antiradical Activity

Structures of luteolinidin, apigeninidin, and HO^•^ were optimized in the Gaussian Program Package 09 [40] at B3LYP/6-311++G(d,p) level of theory, as it was previously shown that this level of theory gives results comparable to the experimental ones for similar compounds. The absence of imaginary frequencies showed that a minima on the energy surface was obtained. The structures of neutral molecules, corresponding radical cations, anions, and radicals were optimized without any geometrical constraints. The experimental environment within the EPR experiment was mimicked by the employed solvent model—Conductor-like Polarizable Continuum model (CPCM) [41]. Natural Bond Orbital (NBO) analysis [42], as implemented in the Gaussian Program Package 09, was utilized to quantify the intramolecular interactions in the obtained radicals and anions.

Reactions between free radicals and radical scavengers can be divided into two main groups: H-atom abstraction and radical adduct formation (RAF) [43,44,45]. The first group includes three different mechanisms depending on the number of steps and the relative order of the electron and proton transfer. The hydrogen atom transfer (HAT) mechanism denotes a fast hydrogen atom transfer from a molecule (ArOH) to a free radical:

ArOH + R^•^ → ArO^•^ + RH (2)

Sequential Proton Loss Electron Transfer (SPLET) is a multi-step mechanism that includes the formation of an anion from a molecule and in the second step formation of a radical and anion from radical, and in the last step protonation of the radical anion:

ArOH → ArO^−^ + H^+^ (3)

ArO^−^ + R^•^ → ArO^•^ + R^−^ (4)

R^−^ + H^+^ → RH (5)

Single Electron Transfer followed by Proton Transfer (SET-PT) is a two-step mechanism consisting for an electron transfer from a molecule and formation of a radical cation, and in the second step proton is transferred to anion formed from a radical:

ArOH + R^•^ → ArOH^•+^ + R^−^ (6)

R^−^ + H^+^ → RH (7)

The change in enthalpy is used to determine the thermodynamically preferred mechanism along with the most reactive positions in the case of polyphenolic compounds. The previously shown reaction steps are characterized by the following changes in the enthalpy, namely bond dissociation enthalpy (ΔH_BDE_), proton affinity (ΔH_PA_), electron transfer enthalpy (ΔH_ETE_), ionization potential (ΔH_IP_), and proton dissociation enthalpy (ΔH_PDE_). The enthalpies of the included species were obtained from the optimized structures at M06-2X/6-311++G(d,p) level of theory.

∆H_BDE_ = H(ArO^•^) + H(RH) − H(ArOH) − H(R^•^) (8)

∆H_PA_ = H(ArO^−^) + H(RH) − H(ArOH) − H(R^−^) (9)

∆H_ETE_ = H(ArO^•^) + H(R^−^) − H(ArO^−^) − H(R^•^) (10)

∆H_IP_ = HArOH^•+^ + H(R^−^) − H(ArOH) − H(R^•^) (11)

∆H_PDE_ = H(ArO^•^) + H(RH) − H(ArOH^•+^) − H(R^−^) (12)

### 2.6. Statistics

Determination of the individual anthocyanins was carried out in triplicate, while all antioxidant tests were performed in duplicate, and all data were expressed as the mean ± standard deviation.

Pattern recognition techniques, such as principal component analysis (PCA) and hierarchic cluster analysis (HCA), were performed using PLS ToolBox, v.6.2.1, for MATLAB 7.12.0 (R2011a), MathWorks, Natick, MA, USA. PCA was carried out as an exploratory data analysis using a singular value decomposition algorithm and a 0.95 confidence level for Q and T^2^ Hotelling limits for outliers.

## 3. Results and Discussion

### 3.1. Phenolic Profile of Analysed Sorghum Grain Samples

**Figure 1:**
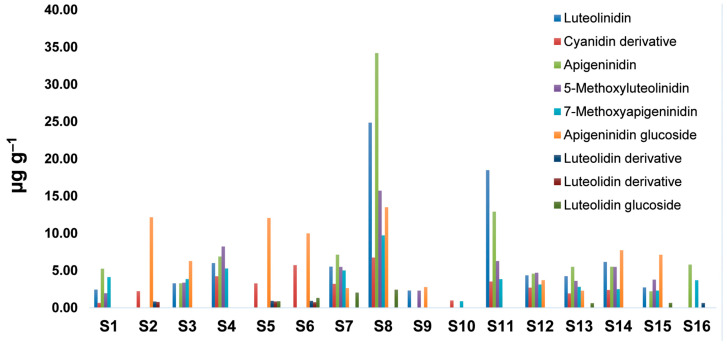
Qualitative and quantitative composition of phenolic compounds present in the tested sorghum grains (µg g^−1^).

Sorghum is attracting increasing attention, not only because it can be grown in drought and high-temperature environments, but also because of its important bioactive compounds [46]. Sorghum is a rare natural source that contains a special type of anthocyanidin, 3-deoxyanthocyanidins, with many potential health benefits [47]. The content of nine phenolic compounds for sorghum grain samples was determined by the HPLC technique (Table S2). The results showed that the composition varied greatly among the analyzed samples (Figure 1). 3-Deoxyanthocyanidins are a special type of anthocyanidin, with many potential health benefits, and their content in sorghum is related to the pericarp color [47]. Apigeninidin and 7-methoxyapigeninidin were the predominant 3-deoxyanthocyanins, accounting for an average of about 40% of the total 3-deoxyanthocyanins in the tested samples. 

The highest number of phenolic compounds was observed in samples **S8** and **S11**, where apigeninidin was recognized as the major metabolite. On the other hand, the lowest amount of phenolic compounds was found in sample **S10**, with low amounts of cyanidin derivatives, and 7-methoxyapigeninidin. Luteolinidin, apigeninidin, apigeninidin glucoside, and 5-methoxyluteolinidin were more abundant compounds in the studied samples compared to other phenolic compounds. Samples **S2**, **S5**, and **S6** showed similar profiles with a high content of apigeninidin glucoside and cyanidin derivatives, while other phenolic compounds were determined in low amounts. Luteolidin glucoside 2 was not detected in samples such as **S1**–**S4**, **S9**–**S12**, **S14**, and **S16**. Total apigeninidin derivatives were the most abundant overall, with the highest level in **S7**. The same sample had the highest concentration of luteolidin derivatives but a low quantity of cyanidin derivatives (Table S2). Due to the effects of different extraction conditions on the amount of extracted 3-deoxyanthocyanins (solvent, extraction time, and temperature), the results obtained in the literature vary widely. In the recent literature, Wu et al. [25] showed a significantly higher content of apigeninidin (329.64 μg g^−1^ to 162.50 μg g^−1^) and luteolinidin (97.18 μg g^−1^ to 82.05 μg g^−1^) in sorghum grains. This could be due to the extraction method, which significantly affects the amount of extracted 3-deoxyanthocyanins [29]. 

### 3.2. Antioxidant Activity 

Since health-related effects are important to consumers, the antioxidant activity of sixteen food-grade sorghum genotypes was evaluated spectrophotometrically using DPPH, ABTS, and FRAP assays, and by electron paramagnetic resonance (EPR) spectroscopy using hydroxyl and ascorbyl radical scavenging assays. The obtained results are presented in Table 1.

The antioxidant potential of the studied sorghum grains was determined by measuring their ability to reduce the 2,2-diphenyl-1-picrylhydrazyl (DPPH) radicals. At the applied concentration of the samples (0.05 g mL^−1^), sample **S10** showed the lowest ability to scavenge DPPH free radicals (0.33 mM TE g^−1^ DW), while the highest ability was observed in sample **S4** (3.62 mM TE g^−1^ DW). 

The results of antioxidant activity in the analyzed samples obtained by the ABTS assay were in agreement with the results obtained by the DPPH assay, where sample **S10** showed the lowest antioxidant capacity, while sample **S4** showed the greatest activity (Table 1). Although there was a strong correlation between the obtained results, the ABTS values were higher than the DPPH values for all samples because the interference of anthocyanins led to an underestimation of antioxidant activity in the DPPH assay [48]. The antioxidant activity data are sometimes hard to compare since there are a couple of different methods and ways of expressing results, which contributes to some discrepancy between the literature data. In recent literature, Kumari et al. [49] showed the highest DPPH and ABTS activities of 14.94 ± 0.18 μg mL^−1^ and 22.84 ± 0.05 μg mL^−1^ observed with IC_50_ value. Furthermore, Ofosu et al. [50] reported DPPH activity of IC_50_ between 499.75 ± 1.49 μg mL^−1^ and 236.0 ± 1.98 μg mL^−1^, while the reported ABTS radical scavenging activity with IC_50_ was from 411.65 ± 1.20 μg mL^−1^ to 317.05 ± 1.06 μg mL^−1^. 

The FRAP method, on the other hand, consists of reducing ferro-tripyridyltriazine complexes (Fe(III)-TPTZ) into intensely blue ferro complexes (Fe(II-TPTZ). In contrast to the DPPH method, antioxidants are able to reduce Fe(III) to Fe(II) ions. The results showed that the antioxidant activity ranged from 0.94 to 3.47 mM TE g^−1^ DW. Sample **S10** demonstrated a significant deviation and unexpected value of antioxidant activity compared with the results obtained by the DPPH and ABTS assays. Moreover, sample **S9** showed the lowest antioxidant capacity, while sample **S4** exhibited the highest antioxidant activity, which is in agreement with the results obtained by the DPPH and ABTS assays (Table 2). 

**Figure 2:**
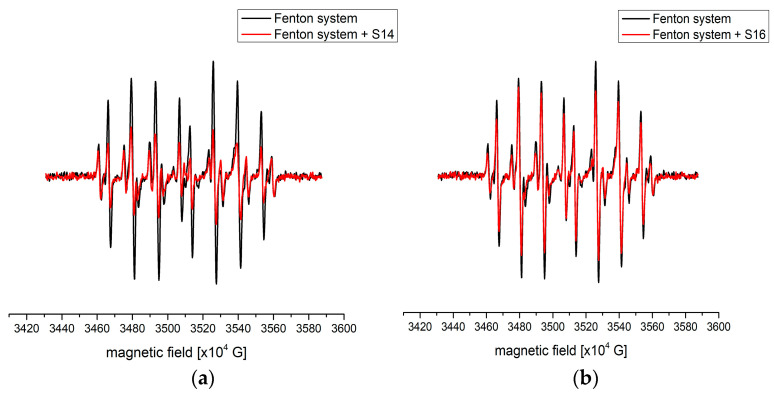

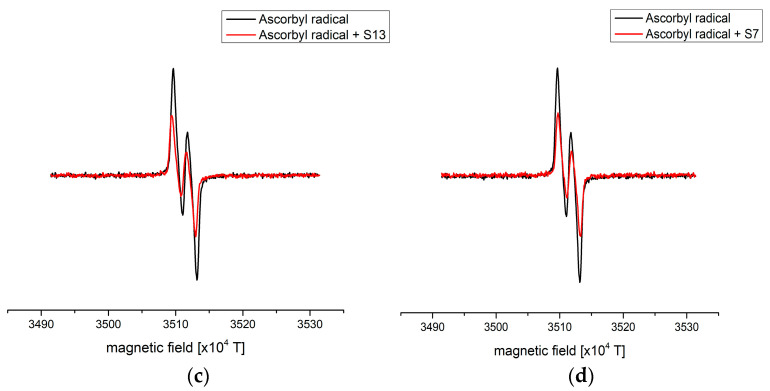
The representative EPR spectra of (**a**) HO^•^ with **S14**, (**b**) HO^•^ with **S16**, (**c**) Asc^•^ with **S13**, and (**d**) Asc^•^ with **S7**.

Hydroxyl (HO^•^) and ascorbyl (Asc^•^) scavenging activity was determined by EPR spectroscopy through direct measurement of the signals belonging to the DEPMPO/HO^•^ and Asc^•^ species. EPR spectra are presented in Figure 2 (representative spectra of samples with the highest and lowest reduction percentages) and Supplementary Material (Figures S1 and S2). The signals used to calculate the reduction percentage are denoted by blue circles in the mentioned figure. The addition of samples in the case of both radicals led to a decrease in signal intensity, giving rise to the conclusion that compounds from samples successfully scavenged radicals. The percentages of scavenged radicals were determined from the decrease in intensity, as explained in the Methodology section.

It is important to mention that the addition of samples into tubes containing the Fenton system led to an increase in signals of other carbon-centered radicals. This change can be observed in Figure 2a,b. In the case of the ascorbyl radical, formation of other radical species was not present. This is expected due to the lower reactivity of this species compared to HO^•^. The complete list of reduction percentages for the investigated samples is presented in Table 1. 

Based on the data in Table 1, it can be concluded that a wide range of reduction percentages of HO^•^ was obtained, between 15.8 (sample **S16**) and 56.6% (sample **S14**). The amount of carbon-centered radical species was higher in the case of sample **S14** than in sample **S16**, proving that a higher amount of organic compounds was present in the former. A certain discrepancy between this test and previously discussed radical scavenging activities is present. This can be attributed to the size and steric hindrance of DPPH and ABTS, which limit the possibility of scavengers reducing the present radical. In the case of **S14**, it was observed that the amount of measured compounds with antioxidant properties was among the highest, which was probably the reason for the calculated percentage. 

On the other hand, the reduction percentages of ascorbyl radicals were in a much narrower range, between 22.0 (sample **S7**) and 42.6% (sample **S13**) (Figure 2c,d, Table 1). The presence of other radical species was not observed due to the lower reactivity of the analyzed system [51]. The phenolic profile of both samples includes luteolinidin, cyaniding, apigeninidin, 5-methoxyluteolinidin, 7-methoxyapigeninidin, and apigeninidin glucoside, which are known as good radical scavengers, but it can be assumed that in the case of sample **S13**, other compounds were also responsible for the scavenging activity. 

### 3.3. Content of the Major and Trace Elements

The presence and concentrations of twenty-two elements were assessed by optical emission spectrometry with inductively coupled plasma (ICP-OES) technique, which provides the possibility of simultaneous determination of a large number of elements present in the samples (Table 2 and Table 3). Nineteen elements were quantified, while three elements, such as Ba, As, and Co, were under the limit of detection. Comparison of the contents of 6 macro- (Ca, K, Mg, Na, P, and S), 13 micro- and trace elements (Fe, Mn, Zn, Al, B, Cd, Co, Cu, Li, Ni, Pb, Se, and Sr) indicate the existence of variation in overall mineral content among the sorghum cultivars and a significant variation in the amount of different elements. Namely, multi-elemental composition can vary depending on the grain’s cultivation conditions and genetic variety. 

Considering the obtained results, the most abundant element was P with concentrations that varied from 3080.63 to 6781.94 mg kg^−1^, which is significantly higher than previously reported data [52,53,54]. A high concentration of P in samples is in accordance with Soetan et al. [55], who remarked that high concentrations of P are important for the structure of carbohydrates and proteins in plants, but also in overall activities of the metabolism, as this element is a part of adenosine triphosphate. The content of measured K was significantly lower compared to the available reports, where K appears as the most abundant element [27,52,53]. Zinc and iron deficiency are major problems worldwide. In some parts of the world, almost every second child suffers from Zn deficiency [56], while Fe deficiency is strongly related to anemia, which affects 60% of children under the age of 5 in poor countries [57]. In addition to children, Fe deficiency is very common among women of reproductive age and lactating mothers [58]. In the present work, the lowest contents of Fe and Zn, with only 13.6 and 13.2 mg kg^−1^, respectively, were detected in **S1**. Considering the obtained results, the amount of these two elements is in agreement with the available data [58] but lower compared to Pontieri et al. [27].

Magnesium is important as a coenzyme in numerous enzymes and therefore human metabolism. In the studied samples, the content of Mg was generally lower compared to results reported by Paiva et al. [52], with the lowest content found in the **S1** sample.

Among the analyzed elements, As, Ba, and Co were below the method quantification limits, while measured concentrations of the least abundant, Li, Sr, Se, Cd, and Ni, were below 0.8 mg kg^−1^ in all studied samples. Concentrations of Pb were below the method quantification limits in all analyzed samples, except in **S3**, with 0.149 mg kg^−1^ of Pb. The content of Al and B ranged from 0.3 to 1.8 mg kg^−1^, while Cu contents ranged from 1.2 (**S1**) to 3.0 mg kg^−1^ (**S16**).

Though required in small quantities in the human body, micronutrients play a significant role in the proper functioning of the body. The presented screening of valuable micronutrients showed that sorghum grain has the potential to provide a significant concentration of minerals in the human diet. 

### 3.4. Nutritional Assessment

Theoretical dietary intakes of major and trace elements in sorghum samples were estimated and compared with the Recommended Daily Allowance (RDA) or Adequate Intake (AI) recommended by the National Institute of Health and the Institute of Medicine [59,60,61,62] for both adult females and males (Table 4). 

Including sorghum in the diet could satisfy a significant contribution to the daily needs of Zn (12–32%), since the RDI value is 8–11 mg/day. With the obtained results, it was noted that the estimated daily intake of 100 g of sorghum would meet the daily Mg requirement between 14 and 29%. Sorghum is not an efficient source of Na and Ca, as it provides below 2% of the RDA and AI. In addition, in all examined samples, K was found at concentrations between 963.54 and 2322.58 mg kg^−1^, which could contribute less than 10% of daily needs. In contrast, it provides significant contents of P, Mn, Fe, Cu, Cr, and Se. Based on the obtained results, sorghum may be recommended as a significant source of P (44–97%) and Se (44–125%) since the intake of about 70 g would completely meet the daily needs of an adult. Selenium content has recently received a lot of attention since selenium is recognized as a cellular antioxidant [63]. Trivalent Cr is known to participate in carbohydrate metabolism and enhance the activity of insulin, which is very suitable for the normal functioning of the body. The obtained results show that 50 g of sorghum could completely satisfy daily needs. Although nutritional assessment may be important, it is crucial to have data on their bioaccessibility.

### 3.5. Multivariate Analysis

#### 3.5.1. PCA and HCA Based on Phenolic Content 

PCA is a commonly used multivariate technique that allows the extraction of full information from a raw data set. Using PCA as a projection method, the initially 9-dimensional data set (9 phenolic compounds) is transformed into 2D coordinates, known as principal components (PCs), using auto-scale as a preprocessing technique. PCA classifies sorghum grain samples based on similarity/dissimilarity, determines samples with different properties from others (outliers), and defines important variables for classification between samples.

**Figure 3:**
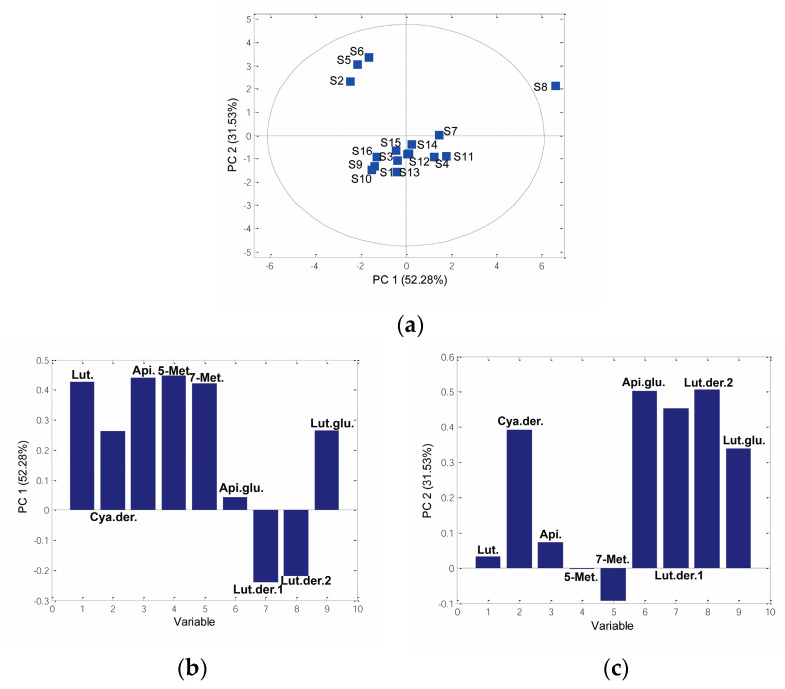
PCA based on sorghum grain species phenolic content: (**a**) 2D score plot PC1 vs. PC2, (**b**) loading plot PC1, (**c**) loading plot PC2. (Lut.—Luteolinidin, Cya. der.—Cyanidin derivative, Api.—Apigeninidin, 5-Met.—5-Methoxyluteolinidin, 7-Met.—7-Methoxyluteolinidin, Api. glu.—Apigeninidin glucoside, Lut. der. 1—Luteolidin derivative 2, Lut. der. 2—Luteolidin derivative 2, Lut. glu.—Luteolidin glucoside).

In the current study, PCA was performed on the data set of phenolic content in sorghum grains. Three principal components (PCs) described 88.99% of the total data variability. PC1 described 52.28% of the variability, while PC2 and PC3 described 31.53% and 5.18%, respectively. Sample **S8** was outside the Hotelling T^2^ 95% probability ellipse and was therefore recognized as an outlier. In contrast to other samples, sample **S8** had the highest amount of luteolinidin, apigeninidin, 5-methoxyluteolinidin, and apigeninidin glucoside. Based on PC2, there were two groups of sorghum genotypes: samples **S2**, **S5**, and **S6** formed one group of samples on the left side of the PCs score plot, while other samples formed a second cluster of samples (Figure 3a). Samples **S2**, **S5**, and **S6** contained a higher amount of apigeninidin glucoside, and a minor amount of luteolidin derivatives 1 and 2, as well as cyanidin derivative**.**

Phenolic compounds, such as luteolinidin, 5-methoxyluteolinidin, 7-methoxyluteolinidin, and apigeninidin, had a positive correlation with PC1, while luteolidin derivatives 1 and 2 showed a negative correlation with PC1 (Figure 3b). According to Figure 3c, PC2 showed a negative correlation with luteolinidin and 7-methoxyapigeninid, while apigeninidin glucoside and luteolin derivative 2 had a positive correlation with PC2.

**Figure 4:**
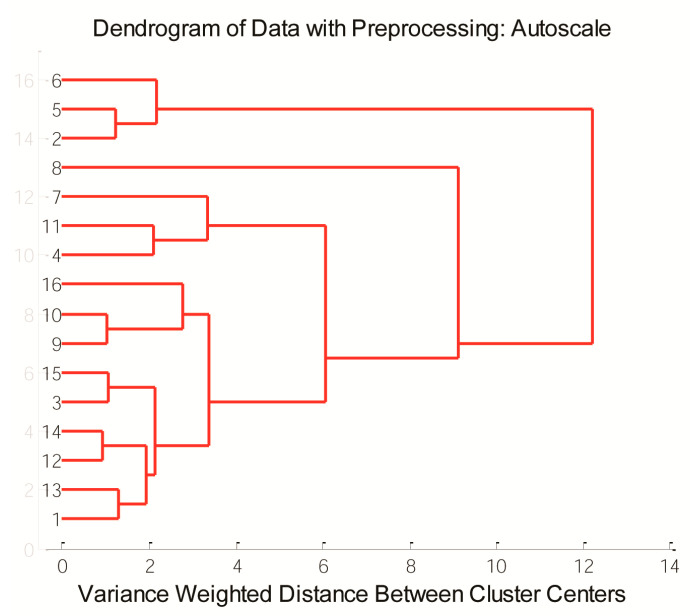
Dendrograms of sorghum grain samples based on phenolic content.

At a 10% similarity level, there were two clusters that differed due to variation between the phenolic content: the first cluster consisted of samples **S2**, **S5**, and **S6** grouped together, while another cluster contained other samples (Figure 4). At a lower similarity level, samples **S4**, **S7**, and **S11** formed one cluster, **S2**, **S5**, and **S6** samples formed a second cluster, **S1**, **S3**, **S9**, **S10**, and **S12**–**S16** formed a third cluster, and samples **S9**, **S10**, and **S16** were grouped together. Due to the highest amount of luteolinidin, apigeninidin, 5-methoxyluteolinidin, and apigeninidin glucoside, sample **S8** was separated from other samples, which is in agreement with PCA analysis.

#### 3.5.2. PCA and HCA Based on Elemental Analysis

**Figure 5:**
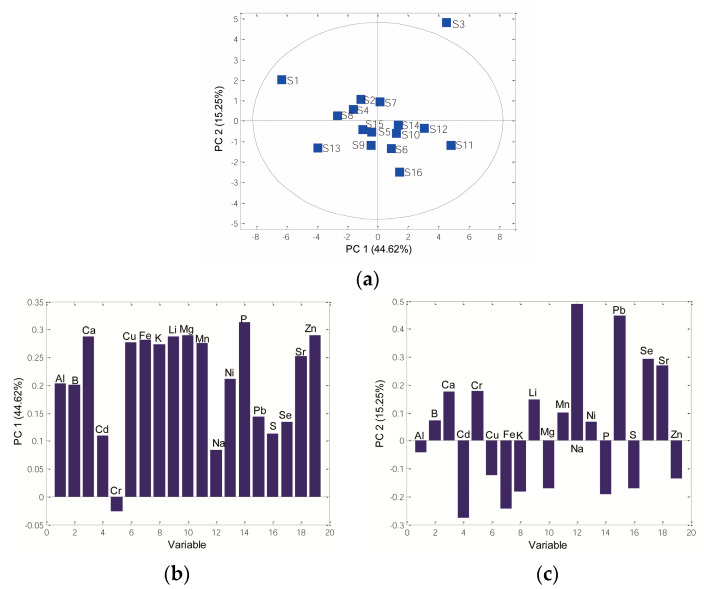
PCA based on element content: (**a**) 2D score plot PC1 vs. PC2, (**b**) loading plot PC1, (**c**) loading plot PC2.

PCA was applied to show the chemical patterns between the analyzed sorghum grains based on elemental profiles. After PCA, three novel PCs described 70.37% of the total data variance. PC1, PC2, and PC3 accounted for 44.62%, 15.25%, and 10.50% of the total variance. One sample, **S3**, was inside the *Hotelling* T^2^ ellipse, suggesting that **S3** was recognized as an outlier. The 2D PCs showed a separation between analysed genotypes, with two groups of samples (Figure 5a): PC1 clearly separated samples **S6**, **S10**, **S11**, **S12**, and **S16** from other samples no. **S2**, **S4**, **S5**, **S7**, **S8**, **S9**, **S13**, and **S15**, while sample **S1** was separated from all investigated samples. Further, according to PC2, there are two groups of samples: samples **S1**, **S2**, **S4**, **S7**, and **S8** formed one cluster, while other samples were positioned on the downside of the PC score plot. 

The loading plots demonstrated the contribution of all analyzed elements to the total variability of the data. The most important elements with the highest contribution on PC1 were Ca, Mg, and P, which agrees with previous studies in which these three metals were found in the highest amount in tested samples. In contrast, Cr negatively contributed to PC1 (Figure 5b). 

PC2 showed a negative loading value for Cd, Fe, K, Mg, and P, while Na, Pb, Cr, Se, and Sr were positively related to PC2 (Figure 5c). Furthermore, Ca, K, Mg, and P were the major elements in the investigated samples, while Cd and Pb were found in the trace. On the other hand, Fe and Se were found to be minor elements.

The HCA divided sorghum samples into groups (clusters) according to similarity and finds the similarity among samples in a multidimensional space, forming clusters between the nearest objects. There are several ways to determine the distance among the samples in a multivariate space, and the best results were obtained using the Ward method. The Euclidean distance for measuring the distance between samples was applied. 

**Figure 6:**
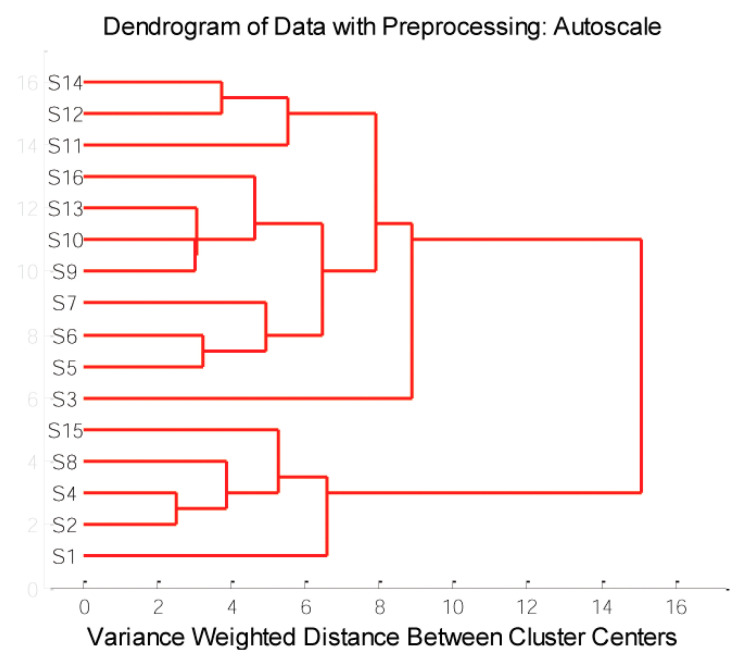
Dendrograms of sorghum grain species samples based on element content.

The HCA considers all the data variability and shows the similarity/dissimilarity among sorghum grains based on element concentration. HCA applied to the 16 samples revealed two clusters at 10 variance-weighted distance units: the first cluster was composed of **S1**, **S2**, **S4**, **S8**, and **S15**, while the second cluster was entirely composed of other samples (Figure 6). The HCA results agreed with those of PCA, in which **S1**, **S2**, **S4**, and **S8** formed one group of samples.

### 3.6. Preferred Anti-HO^•^ Scavenging Mechanism of Luteolinidin and Apigeninidin

**Figure 7:**
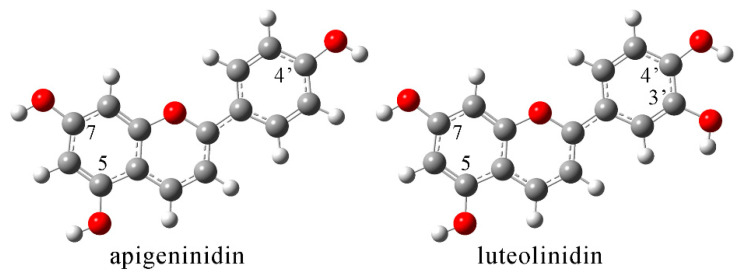
Optimized structures (at B3LYP/6-311++G(d,p) level of theory) of apigeninidin and luteolinidin.

As previously discussed, luteolinidin and apigeninidin are polyphenols characteristic of sorghum and are among the most important compounds for antioxidant activity. The EPR spectroscopy measurements showed a significant reduction in the ability of the extracts towards HO^•^. Therefore, it is beneficial to determine the thermodynamically preferred mechanism of the anti-radical activity of these two compounds towards HO^•^. The optimized structures (at B3LYP/6-311++G(d,p) level of theory) of luteolinidin and apigeninidin are presented in Figure 7 with the enumeration scheme.

The structures of apigeninidin and luoteolinidin contain several OH groups that are the most reactive parts of a molecule when it comes to radical scavenging activity. Both compounds are characterized by OH groups in positions 5 and 7 of benzopyrylium rings. Luteolinidin has a catechol moiety as part of the structure, while apigeninidin has one OH group less on the aryl ring. Both compounds are positively charged with a planar structure due to the delocalization of charge. Table 5 lists the thermodynamic parameters calculated according to Equations (7)–(9).

The HAT mechanism is exothermic in all of the investigated positions for both compounds. The ΔH_BDE_ are the same in positions 5 and 7 (−127 and −115 kJ mol^−1^), proving that the aromatic ring substituents do not influence the spontaneity of the process. The hydrogen atom loss from OH groups that are part of benzopyrylium rings leads to spin density distribution throughout the whole system with delocalized electron density. The hydrogen atom transfer from the OH group in position 4′ of apigeninidin is less exothermic (−125 kJ mol^−1^) than from the OH group in position 5. On the other hand, the presence of catechol moiety significantly increases the proton [64] donating ability of luteolinidin, with ΔH_BDE_ being −147 (position 3′) and −149 kJ mol^−1^ (position 4′). The calculated values reproduce well the ones obtained by Ali and Ali [65]. The intramolecular hydrogen bond formed between adjacent OH groups increases the overall stability of the formed radical. The stabilization interactions formed between these two groups had stabilization energy of 1.05 and 1.07 kJ mol^−1^, as calculated in the NBO analysis. When changes in enthalpy of the first steps of the investigated mechanisms are compared, it can be concluded that the least thermodynamically preferred mechanism is SPLET, with the ΔH_IP_ values being positive for both compounds (287 kJ mol^−1^ for apigeninidin and 276 kJ mol^−1^ for luteolinidin). The starting structures of both compounds were positively charged, and it can be assumed that the loss of an electron in the first step of the SET-PT mechanism would destabilize the system. The proton transfer, as the second step of SET-PT, is highly exothermic and, therefore, never a limiting factor. The most exothermic first step of the analyzed mechanisms is proton exchange in the SPLET mechanisms. Upon proton removal, a neutral species is formed from the investigated compounds. Again, the ΔH_PA_ are the same for OH groups in positions 5 and 7 of both compounds (−295 kJ mol^−1^). The proton removal from the OH group in position 4′ of apigeninidin has slightly lower exothermicity (−284 kJ mol^−1^). Two OH groups of the catechol moiety of luteolinidin have different abilities to donate protons (−262 for position 3′ and −301 kJ mol^−1^ for position 4′). The hydrogen bonds are again formed between negatively charged oxygen and OH groups, with energies of 4.07 and 5.8 for positions 3′ and 4′, respectively. The difference is due to the position of the group, as the para-substitution allows easier distribution of charge throughout the aromatic ring. The planarity of the system in obtained anions is preserved, which allows better delocalization within the system. The second step of the SPLET process includes electron transfer, which is usually a diffusion-controlled process. Positions 5 and 7 of apigeninidin and position 4′ of luteolinidin are the most reactive positions when HO^•^ scavenging activity is concerned. It should be mentioned that other mechanisms, such as radical adduct formation or combined processes, are possible [44,66], but they are beyond the scope of this article. The presence of the catechol moiety marks luteolinidin as a much more potent radical scavenger compared to apigeninidin. The addition of glucoside influences the stability, although one OH group is less available for radical scavenging and, therefore, lowers the antiradical potency. 

## 4. Conclusions

In summary, the polyphenolic profile, macro-, micro-, and trace element content, as well as the antioxidant potential of sixteen sorghum grain genotypes grown under agroecological conditions in Serbia, were studied. A significant content of 3-deoxyanthocyanins (apigeninidin, luteolinidin, and their derivatives) was found, which contributes greatly to the total antioxidant capacity. The application of the DPPH, ABTS, hydroxyl, and ascorbyl radical scavenging, and FRAP assays showed that the studied sorghum grains have promising antioxidant potential. Multi-elemental analysis of the samples showed the presence of a significant amount of minerals for human nutrition. Moreover, the estimated dietary intake of P, Mg, Mn, Cu, Zn, and Se from the consumption of 100 g of the tested grains significantly fulfills the recommended nutritional values for adult females and males. Based on pattern recognition techniques, samples with a high content of phenolic compounds were identified, and these samples could be breeding materials for further development of sorghum variety as a promising source of bioactives. The theoretical analysis of anti-radical activity pointed out Sequential Proton Loss Electron Transfer as the thermodynamically preferred mechanism of apigeninidin and luteolinidin. The presence of intramolecular hydrogen bonds within the structure of the radical formed increased the radical scavenging potency of luteolinidin compared to apigeninidin. The results suggest that sorghum grains may be an effective material for functional foods that provide many human health benefits.

## Figures and Tables

**Table 1:** Antioxidant activity of the tested sorghum grains (mean ± SD of dry weight of the sample).

Sample	DPPH (mm TE * g^−1^)	FRAP (mm TE g^−1^)	ABTS (mm TE g^−1^)	HO^•^ (%)	Asc^•^ (%)
S1	0.61 ± 0.05	1.01 ± 0.07	0.95 ± 0.11	42.10 ± 0.14	38.40 ± 0.28
S2	1.46 ± 0.05	1.92 ± 0.00	2.80 ± 0.01	27.45 ± 0.21	32.15 ± 0.21
S3	0.58 ± 0.00	0.99 ± 0.03	1.14 ± 0.08	39.85 ± 0.21	40.55 ± 0.35
S4	3.62 ± 0.05	3.47 ± 0.05	4.17 ± 0.01	45.50 ± 0.57	36.25 ± 0.35
S5	2.09 ± 0.07	2.48 ± 0.03	3.17 ± 0.08	26.05 ± 0.21	30.60 ± 0.42
S6	3.75 ± 0.00	3.41 ± 0.08	4.16 ± 0.02	39.35 ± 0.35	35.45 ± 0.35
S7	3.05 ± 0.08	3.04 ± 0.01	3.58 ± 0.08	36.95 ± 0.35	22.00 ± 0.42
S8	3.06 ± 0.12	3.19 ± 0.01	3.85 ± 0.03	32.4 ± 0.42	34.50 ± 0.42
S9	0.36 ± 0.02	0.94 ± 0.02	0.76 ± 0.04	26.1 ± 0.42	37.45 ± 0.49
S10	0.33 ± 0.03	1.12 ± 0.11	0.62 ± 0.03	16.55 ± 0.35	35.45 ± 0.49
S11	3.51 ± 0.01	3.25 ± 0.08	3.84 ± 0.10	28.25 ± 0.35	40.25 ± 0.35
S12	3.03 ± 0.18	2.94 ± 0.05	3.71 ± 0.07	26.9 ± 0.28	34.35 ± 0.35
S13	2.43 ± 0.14	2.70 ± 0.05	3.22 ± 0.01	27.85 ± 0.35	42.60 ± 0.42
S14	1.68 ± 0.01	2.20 ± 0.10	2.66 ± 0.03	56.55 ± 0.35	37.45 ± 0.35
S15	0.57 ± 0.01	1.08 ± 0.02	1.07 ± 0.05	37.35 ± 0.21	32.15 ± 0.49
S16	0.37 ± 0.01	0.95 ± 0.06	0.71 ± 0.01	15.80 ± 0.28	40.35 ± 0.35

* TE—Trolox-equivalent.

**Table 2:** Content of macroelements (mg kg^−1^ ± SD) in the tested sorghum grains.

Sample	Ca	K	Mg	Na	P	S
S1	98.26 ± 3.51	963.30 ± 20.12	575.81 ± 16.37	4.59 ± 0.24	3080.87 ± 22.17	881.32 ± 5.80
S2	151.20 ± 1.64	2055.46 ± 14.65	786.71 ± 7.33	3.91 ± 0.03	5375.47 ± 17.44	772.15 ± 0.70
S3	189.93 ± 2.86	1951.90 ± 12.12	835.95 ± 11.79	5.39 ± 0.04	6069.05 ± 3.37	849.05 ± 1.35
S4	141.78 ± 0.61	1709.71 ± 26.67	816.78 ± 17.55	3.24 ± 0.02	5196.2 ± 10.13	897.46 ± 2.03
S5	145.70 ± 1.18	1961.74 ± 4.37	776.14 ± 0.00	3.51 ± 0.03	5320.82 ± 16.8	760.46 ± 0.00
S6	163.49 ± 1.00	2249.17 ± 18.89	812.45 ± 5.49	2.96 ± 0.10	5917.35 ± 3.43	780.40 ± 0.69
S7	146.63 ± 1.55	1586.28 ± 9.31	840.57 ± 2.41	4.39 ± 0.03	5123.39 ± 3.45	837.89 ± 0.00
S8	113.99 ± 2.73	1700.97 ± 22.39	790.87 ± 2.45	3.01 ± 0.04	5051.45 ± 20.99	725.31 ± 0.70
S9	123.79 ± 0.37	1638.96 ± 17.85	856.75 ± 5.73	2.97 ± 0.02	5561.64 ± 23.58	893.67 ± 1.35
S10	134.58 ± 3.44	2133.49 ± 5.62	888.96 ± 7.02	3.69 ± 0.06	6163.09 ± 7.02	895.91 ± 0.70
S11	174.06 ± 0.21	2233.90 ± 32.42	927.35 ± 7.05	3.64 ± 0.09	6781.94 ± 28.19	1030.00 ± 2.11
S12	183.20 ± 1.65	2322.58 ± 0.00	878.04 ± 4.71	4.23 ± 0.04	6342.75 ± 10.09	817.40 ± 1.68
S13	135.75 ± 2.42	1818.34 ± 21.11	841.49 ± 4.15	3.40 ± 0.02	5759.25 ± 17.31	807.71 ± 2.08
S14	161.11 ± 3.20	1985.60 ± 39.78	823.80 ± 5.39	3.45 ± 0.04	5792.33 ± 13.48	859.55 ± 0.00
S15	80.11 ± 0.07	1627.88 ± 27.89	734.82 ± 9.41	2.99 ± 0.03	4791.95 ± 10.46	853.63 ± 3.14
S16	119.50 ± 0.31	1994.87 ± 29.45	831.32 ± 1.03	3.16 ± 0.10	6322.65 ± 30.82	1031.82 ± 4.45

**Table 3:** Content of microelements (mg kg^−1^ ± SD) in the tested sorghum grains.

Sample	Al	B	Cd	Cr	Cu	Li	Ni	Pb	Se	Sr	Mn	Zn	Fe
S1	0.33 ± 0.10	0.58 ± 0.00	<0.005	0.15 ± 0.00	1.20 ± 0.03	0.06 ± 0.00	0.25 ± 0.02	<0.005	0.32 ± 0.03	0.10 ± 0.00	4.65 ± 0.18	13.16 ± 0.17	13.60 ± 0.62
S2	0.63 ± 0.22	0.44 ± 0.03	<0.005	0.47 ± 0.23	2.13 ± 0.02	0.09 ± 0.00	0.25 ± 0.02	<0.005	0.38 ± 0.04	0.15 ± 0.00	6.80 ± 0.19	18.23 ± 0.09	18.79 ± 0.65
S3	1.13 ± 0.14	0.69 ± 0.01	0.01 ± 0.00	0.29 ± 0.17	2.83 ± 0.01	0.11 ± 0.01	0.65 ± 0.00	0.15 ± 0.06	0.69 ± 0.22	0.21 ± 0.00	13.05 ± 0.13	23.55 ± 0.02	23.44 ± 0.18
S4	0.48 ± 0.15	0.33 ± 0.01	<0.005	0.37 ± 0.12	1.74 ± 0.03	0.08 ± 0.00	0.33 ± 0.00	<0.005	0.38 ± 0.05	0.16 ± 0.00	7.99 ± 0.16	17.21 ± 0.00	18.39 ± 0.31
S5	1.43 ± 0.21	0.38 ± 0.02	0.02 ± 0.00	0.13 ± 0.01	2.07 ± 0.00	0.09 ± 0.00	0.16 ± 0.00	<0.005	0.36 ± 0.10	0.14 ± 0.00	6.68 ± 0.08	17.40 ± 0.02	20.60 ± 0.76
S6	0.78 ± 0.15	0.27 ± 0.01	0.03 ± 0.00	0.17 ± 0.01	2.73 ± 0.02	0.10 ± 0.00	0.17 ± 0.01	<0.005	0.39 ± 0.07	0.16 ± 0.01	8.53 ± 0.04	20.76 ± 0.00	23.30 ± 0.15
S7	1.65 ± 0.10	0.52 ± 0.01	0.02 ± 0.00	0.14 ± 0.01	2.01 ± 0.03	0.09 ± 0.00	0.61 ± 0.00	<0.005	0.34 ± 0.23	0.15 ± 0.01	10.56 ± 0.02	15.68 ± 0.02	19.85 ± 0.18
S8	0.87 ± 0.01	0.57 ± 0.02	<0.005	0.57 ± 0.08	1.87 ± 0.04	0.07 ± 0.00	0.39 ± 0.01	<0.005	0.40 ± 0.02	0.09 ± 0.00	7.57 ± 0.07	15.55 ± 0.04	19.96 ± 0.06
S9	0.42 ± 0.43	0.53 ± 0.01	0.01 ± 0.01	0.25 ± 0.14	2.51 ± 0.03	0.08 ± 0.00	0.29 ± 0.00	<0.005	0.38 ± 0.01	0.12 ± 0.00	10.16 ± 0.01	19.88 ± 0.04	24.94 ± 0.20
S10	1.05 ± 0.20	0.61 ± 0.02	<0.005	0.28 ± 0.06	2.67 ± 0.02	0.08 ± 0.01	0.32 ± 0.00	<0.005	0.41 ± 0.02	0.14 ± 0.01	9.71 ± 0.01	20.81 ± 0.01	27.16 ± 0.50
S11	1.69 ± 0.10	1.07 ± 0.03	0.02 ± 0.00	0.09 ± 0.02	2.34 ± 0.02	0.10 ± 0.00	0.71 ± 0.01	<0.005	0.46 ± 0.06	0.15 ± 0.01	12.03 ± 0.12	24.20 ± 0.04	33.74 ± 0.35
S12	1.81 ± 0.03	0.90 ± 0.02	0.01 ± 0.00	0.34 ± 0.03	2.42 ± 0.03	0.11 ± 0.00	0.62 ± 0.00	<0.005	0.24 ± 0.06	0.15 ± 0.00	8.48 ± 0.03	19.70 ± 0.04	30.82 ± 0.03
S13	0.75 ± 0.16	0.56 ± 0.03	<0.005	<0.005	2.19 ± 0.02	0.09 ± 0.00	0.39 ± 0.01	<0.005	0.34 ± 0.04	0.13 ± 0.01	8.03 ± 0.15	19.91 ± 0.03	23.71 ± 0.77
S14	1.10 ± 0.09	0.97 ± 0.02	0.02 ± 0.01	0.43 ± 0.03	2.60 ± 0.00	0.09 ± 0.00	0.84 ± 0.00	<0.005	0.31 ± 0.05	0.16 ± 0.01	6.69 ± 0.01	20.89 ± 0.08	21.06 ± 0.31
S15	1.06 ± 0.19	0.23 ± 0.02	0.02 ± 0.00	0.18 ± 0.07	1.64 ± 0.00	0.05 ± 0.00	0.37 ± 0.00	<0.005	0.41 ± 0.07	0.06 ± 0.00	6.27 ± 0.01	16.42 ± 0.04	19.01 ± 0.23
S16	0.87 ± 0.08	0.44 ± 0.02	0.02 ± 0.00	0.08 ± 0.03	3.05 ± 0.00	0.08 ± 0.00	0.54 ± 0.01	<0.005	0.40 ± 0.14	0.12 ± 0.01	9.08 ± 0.07	25.45 ± 0.10	27.45 ± 0.52

Concentration of As, Ba and Co were under the limit of detection (<0.005) in all tested samples.

**Table 4:** Daily intake estimations of selected macro- and microelements through the consumption of 100 g of tested sorghum grains.

Analyte	Daily Intake mg/100 g Grain	RDA/AI * (F) (mg/day)	RDA/AI * (M) (mg/day)	% Intake (F)	% Intake (M)
Mg	57.58–92.74	320	420	18–29	14–22
P	308.06–678.19	700	700	44–97	44–97
K	96.35–232.26	2600	3400	4–9	3–7
Ca	8.01–19.00	1000	1000	1–2	1–2
Na	0.30–0.54	1500 *	1500 *	0	0
Mn	0.47–1.31	1.80 *	2.30 *	26–73	20–57
Fe	1.36–3.37	18.00	8.00	8–19	17–42
Cu	0.12–0.30	0.90	0.90	13–33	13–33
Zn	1.32–2.54	8.00	11.00	17–32	12–23
Cr	0.01–0.06	0.025 *	0.035 *	32–228	23–163
Se	0.02–0.07	0.055	0.055	44–125	44–125

* AI: Adequate Intake; RDA: Recommended Daily Allowance; M: male 31–50 years old; F: female 31–50 years old.

**Table 5:** The change in reaction enthalpies for the common mechanisms (in kJ mol^−1^) of apigeninidin and luteolinidin.

Compound	Position	HAT	SPLET		SET-PT	
		ΔH_BDE_	ΔH_PA_	ΔH_ETE_	ΔH_IP_	ΔH_PDE_
	5	−127	−295	167	287	−415
apigeninidin	7	−115	−295	180	287	−402
	4′	−125	−284	159	287	−413
luteolinidin	5	−127	−295	168	276	−403
	7	−115	−295	180	276	−391
	3′	−147	−262	115	276	−423
	4′	−149	−301	152	276	−425

## Data Availability

Data supporting the obtained results can be obtained from the authors upon request.

## Supplementary Materials

The following supporting information can be downloaded at: https://www.mdpi.com/article/10.3390/antiox12081485/s1, Table S1: The list of sixteen food grade sorghum genotypes within IFVCNS (Institute of Field and Vegetable Crops, Novi Sad, National Institute of the Republic of Serbia) collection; Table S2: Qualitative and quantitative composition of phenolic compounds present in tested sorghum grains (µg g^−1^ ± SD); Figure S1: The EPR spectra of hydroxyl radical with samples **S1**–**S16**; Figure S2: The EPR spectra of ascorbyl radical with samples **S1**–**S16**.
