# Supplementary material for: Chemical Composition, Antioxidant Potential, and Nutritional Evaluation of Cultivated Sorghum Grains: A Combined Experimental, Theoretical, and Multivariate Analysis
Source: Antioxidants (Basel). 2023 Jul 25;12(8):1485. doi: 10.3390/antiox12081485 (PMC10451854; doi:10.3390/antiox12081485)
Supplement: Supplementary file 1 [file antioxidants-12-01485-s001.zip › antioxidants-2509323-supplementary.pdf]

SUPPLEMENTARY MATERIAL for

Chemical composition, antioxidant potential, and nutritional evaluation of cultivated sorghum grains: Combined experimental, theoretical, and multivariate analysis

By the authors: Simona Jaćimović, Biljana Kiprovski, Petar Ristivojević, Dušan Dimić, Đura Nakarada, Biljana Dojčinović, Vladimir Sikora, Nemanja Teslić and Nebojša Đ. Pantelić

Table S1. The list of sixteen food grade sorghum genotypes within IFVCNS (Institute of Field and Vegetable Crops, Novi Sad, National Institute of the Republic of Serbia) collection.

Analyte	Sample number	Genotype	Original collection
**S1**	Re2	AK 3001	Gabonatermesztesi Kutato Intezet Szeged, HUN
**S2**	Re31	ROKY 78	Oklahoma State University Stillwater, USA
**S3**	Re45	NK R 310	Gabonatermesztesi Kutato Intezet Szeged, HUN
**S4**	Re161	ROKY 27	Oklahoma State University Stillwater, USA
**S5**	Re168	ROKY 78	Oklahoma State University Stillwater, USA
**S6**	Re169	ROKY 78	Oklahoma State University Stillwater, USA
**S7**	Re187	IA 14	Iowa State University Ames, USA
**S8**	Re192	IA 19	Iowa State University Ames, USA
**S9**	Re218	RTx 434	Texas A&M University College Station, USA
**S10**	Re230	RTx 2744	Texas Agric. Experiment Station Lubbock, USA
**S11**	Re248	32612 T	Unknown
**S12**	Re258	NK 115 978/90	Northrup King, USA
**S13**	Re261	NK 90 970/90	Northrup King, USA
**S14**	Re262	Pioneer 8910 968/90	Pioneer, USA
**S15**	Re279	Tx 2834	Texas A&M University College Station, USA
**S16**	Re284	Tx 2839	Texas A&M University College Station, USA

Table S2. Qualitative and quantitative composition of phenolic compounds present in tested Sorghum grains (μg g^{-1} ± SD).

Sample	Luteolinidin	Cyanidin derivative	Apigeninidin	5-methoxyluteolinidin	7-methoxyapigeninidin	Apigeninidin glucoside	Luteolidin derivative 1	Luteolidin derivative 2	Luteolidin glucoside
**S1**	2.44±0.00	0.65±0.13	5.24±0.12	1.94±0.10	4.12±0.11	-	-		-
**S2**	-	2.22±0.10	-	-	-	12.15±0.22	0.84±0.07	0.78±0.07	-
**S3**	3.28±0.00	-	3.28±0.00	3.36±0.65	3.85±0.02	6.28±0.57	-	-	-
**S4**	5.99±0.11	4.22±0.32	6.88±0.35	8.23±0.25	5.28±0.22	-	-	-	-
**S5**	-	3.26±0.31	-	-	-	12.06±0.62	0.92±0.06	0.84±0.08	0.87±0.08
**S6**	-	5.72±0.10	-	-	-	9.98±0.06	0.93±0.07	0.74±0.09	1.32+0.21
**S7**	5.52±0.42	3.20±0.24	7.13±0.02	5.47±0.38	5.02±0.10	2.64±0.07	-	-	2.03±0.03
**S8**	24.85±1.42	6.74±0.17	34.20±3.65	15.72±1.39	9.72±0.09	13.49±0.64	-	-	2.42±0.09
**S9**	2.31±0.08	-	-	2.30±0.08	-	2.77±0.22	-	-	-
**S10**	-	0.97±0.08	-	-	0.88±0.14	-	-	-	-
**S11**	18.47±0.23	3.51±0.20	12.90±0.29	6.27±0.05	3.84±0.08	-	-	-	-
**S12**	4.35±0.07	2.70±0.28	4.57±0.43	4.70±0.02	3.13±0.14	3.70±0.25	-	-	-
**S13**	4.24±0.38	1.91±0.07	5.50±0.33	3.60±0.33	2.81±0.47	2.28±0.21	-	-	0.62±0.02
**S14**	6.15±0.15	2.39±0.27	5.51±0.42	5.48±0.09	2.50±0.43	7.72±0.66	-	-	-
**S15**	2.72±0.20	-	2.20±0.13	3.76±0.22	2.31±0.06	7.12±0.02	-		0.65±0.02
**S16**	-	-	5.80±0.90	-	3.69±0.46	-	0.64±0.05	-	-

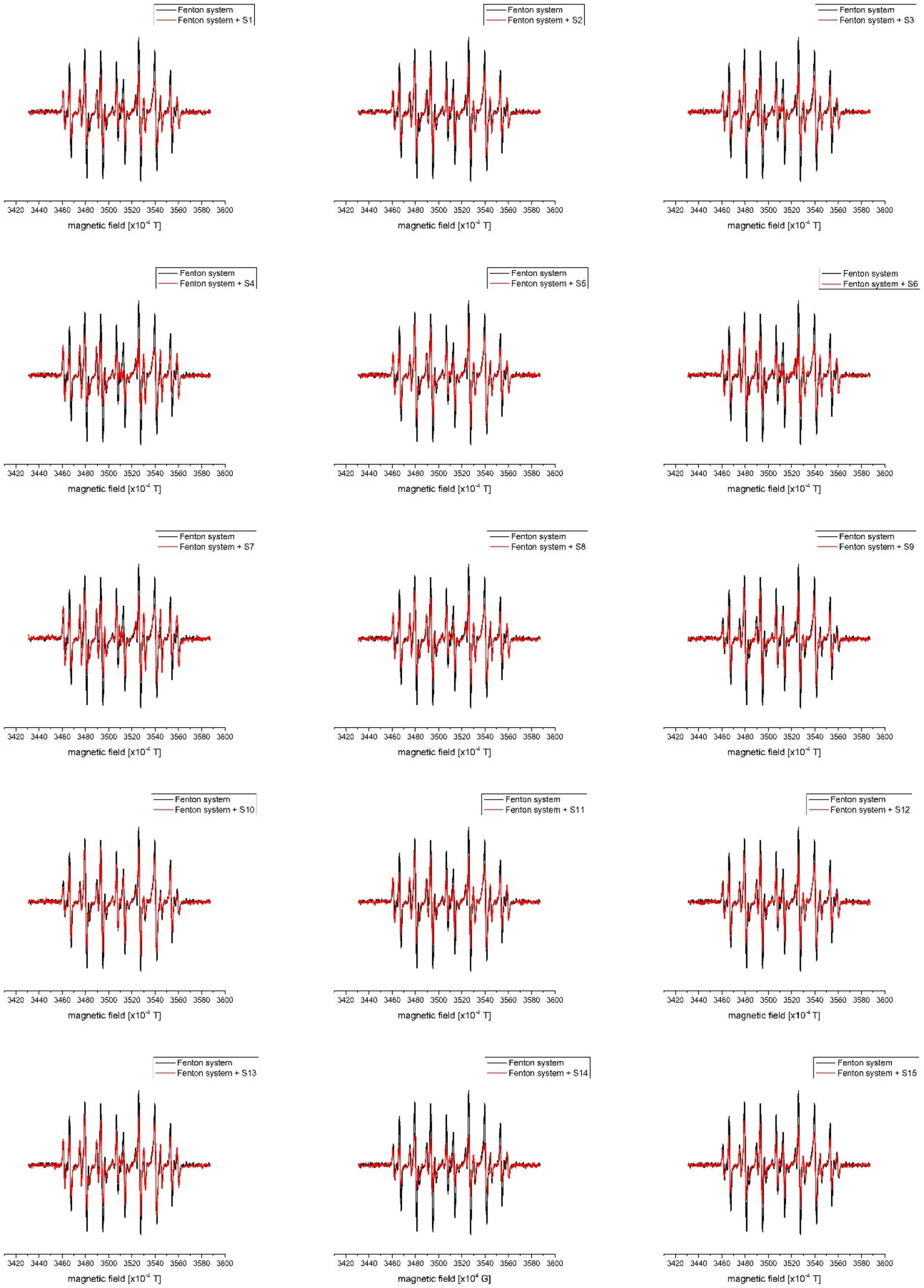
Fenton system
Fenton system + S1
3420 3440 3460 3480 3500 3520 3540 3560 3580 3600
magnetic field [x10^{-4} T]
Fenton system
Fenton system + S2
3420 3440 3460 3480 3500 3520 3540 3560 3580 3600
magnetic field [x10^{-4} T]
Fenton system
Fenton system + S3
3420 3440 3460 3480 3500 3520 3540 3560 3580 3600
magnetic field [x10^{-4} T]
Fenton system
Fenton system + S4
3420 3440 3460 3480 3500 3520 3540 3560 3580 3600
magnetic field [x10^{-4} T]
Fenton system
Fenton system + S5
3420 3440 3460 3480 3500 3520 3540 3560 3580 3600
magnetic field [x10^{-4} T]
Fenton system
Fenton system + S6
3420 3440 3460 3480 3500 3520 3540 3560 3580 3600
magnetic field [x10^{-4} T]
Fenton system
Fenton system + S7
3420 3440 3460 3480 3500 3520 3540 3560 3580 3600
magnetic field [x10^{-4} T]
Fenton system
Fenton system + S8
3420 3440 3460 3480 3500 3520 3540 3560 3580 3600
magnetic field [x10^{-4} T]
Fenton system
Fenton system + S9
3420 3440 3460 3480 3500 3520 3540 3560 3580 3600
magnetic field [x10^{-4} T]
Fenton system
Fenton system + S10
3420 3440 3460 3480 3500 3520 3540 3560 3580 3600
magnetic field [x10^{-4} T]
Fenton system
Fenton system + S11
3420 3440 3460 3480 3500 3520 3540 3560 3580 3600
magnetic field [x10^{-4} T]
Fenton system
Fenton system + S12
3420 3440 3460 3480 3500 3520 3540 3560 3580 3600
magnetic field [x10^{-4} T]
Fenton system
Fenton system + S13
3420 3440 3460 3480 3500 3520 3540 3560 3580 3600
magnetic field [x10^{-4} T]
Fenton system
Fenton system + S14
3420 3440 3460 3480 3500 3520 3540 3560 3580 3600
magnetic field [x10^{4} G]
Fenton system
Fenton system + S15
3420 3440 3460 3480 3500 3520 3540 3560 3580 3600
magnetic field [x10^{-4} T]

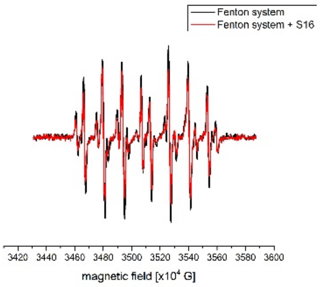

Figure S1. The EPR spectra of hydroxyl radical with samples **S1–S16**.

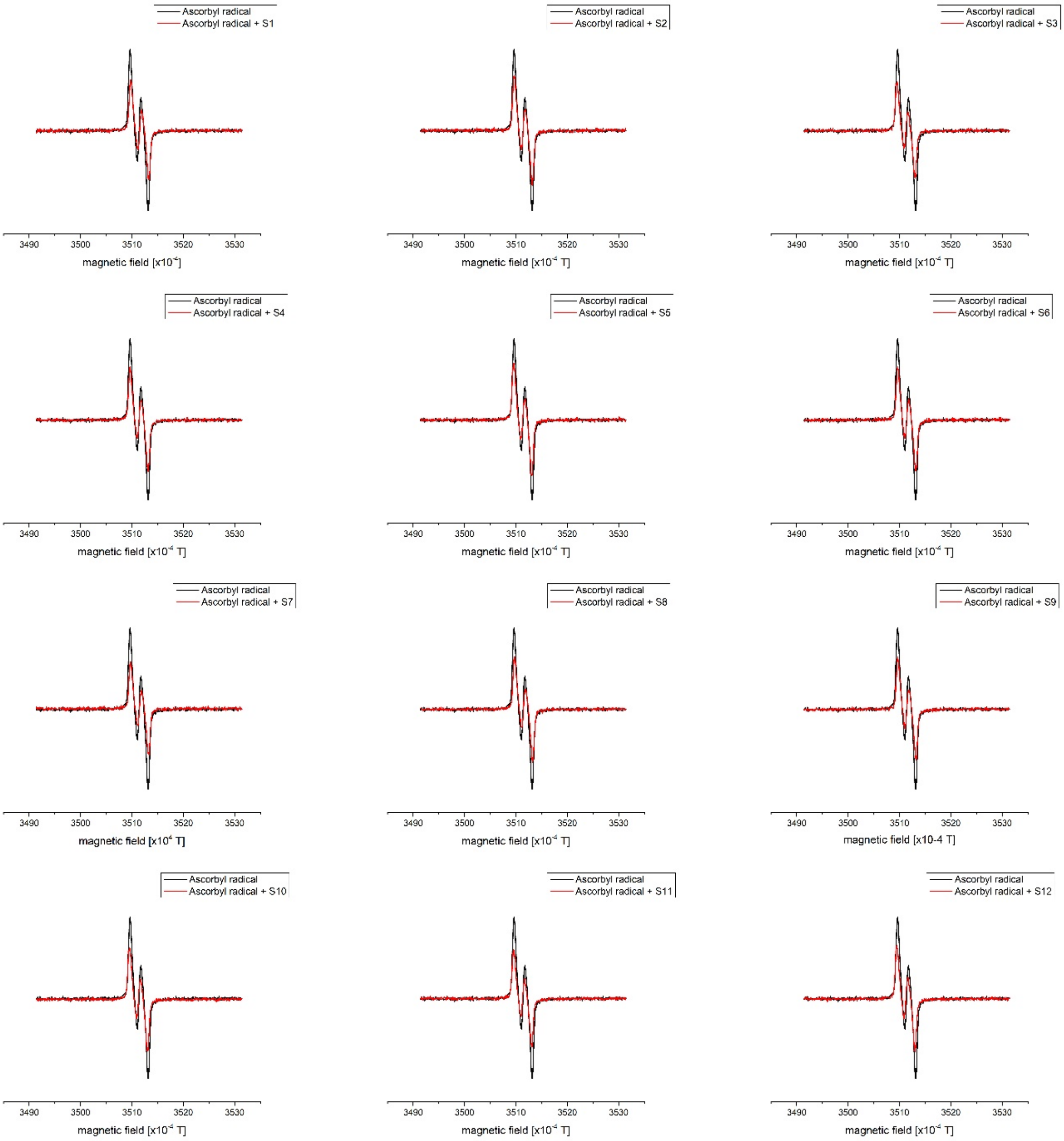

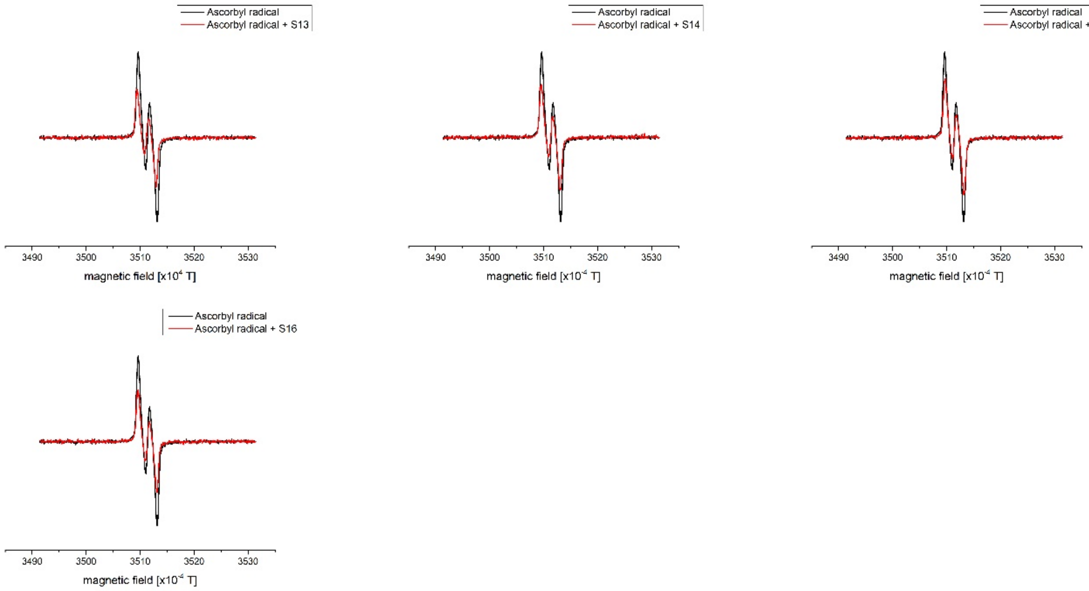

Figure S2. The EPR spectra of ascorbyl radical with samples **S1–S16**.
